# Colon Capsule Endoscopy in the Routine Diagnostic Pathway for Colorectal Diseases (DanCap): Protocol for a Cluster-Allocated Crossover Trial

**DOI:** 10.3390/diagnostics16162657

**Published:** 2026-08-20

**Authors:** Alexandra Agache, Lasse Kaalby Møller, Sebastian Radic Eskemose, Ola Selnes, Ulrik Deding, Thomas Bjørsum-Meyer, Issam Al-Najami, Anders Høgh, Benedicte Schelde-Olesen, Charlotte Løfberg, Tue Kjølhede, Maja Kjær Rasmussen, Gunnar Baatrup, Anastasios Koulaouzidis

**Affiliations:** 1 Department of Clinical Research, University of Southern Denmark, 5230 Odense, Denmark; alexandra.agache@rsyd.dk (A.A.); lasse.kaalby.moller@rsyd.dk (L.K.M.);; 2 Department of Surgery, Odense University Hospital, 5000 Odense, Denmark; charlotte.loefberg@rsyd.dk; 3 Department of General Surgery, Carol Davila University of Medicine and Pharmacy, 050474 Bucharest, Romania; 4 Centre for Innovative Medical Technology, Odense University Hospital, 5000 Odense, Denmark

**Keywords:** colon capsule endoscopy, colonoscopy, cost–consequence analysis, diagnostic pathway, cluster-allocated crossover, protocol

## Abstract

**Background/Objectives**: Colonoscopy is the standard examination for many symptomatic patients referred for lower-gastrointestinal investigation, but it is resource-intensive and may be a burdensome experience. Colon capsule endoscopy (CCE) offers a minimally invasive, sedation-free first-line examination, although clinically important findings, inadequate cleansing or incomplete transit can generate downstream colonoscopy. DanCap aims to compare the costs and clinical consequences of a CCE-first pathway with routine conventional colonoscopy (CC). **Methods**: DanCap is a single-centre, cluster-allocated crossover trial at Odense University Hospital, Denmark. General practice clinics follow CCE or CC according to the parity of their pre-existing provider number, with pathways crossing after 200 consecutive CCE participants; no trial-generated random allocation sequence is used. Eight hundred symptomatic adults aged >18 years referred for expedited lower-gastrointestinal investigation are planned. CCE participants with suspected cancer, any polyp ≥6 mm, inadequate cleansing or an incomplete examination are referred for colonoscopy. The primary outcome is pathway cost. Registered secondary outcomes and prespecified process measures include polyp and colorectalcancer detection, examination quality, reinvestigation and patient-reported consequences. FIT and microbiome are registered secondary outcomes; participation in the substudy is optional and the analyses are exploratory within the CCEpathway. Primary analyses will follow the assigned pathway and account for GP clinic clustering, period and allocation sequence. **Expected Results**: The trial will quantify the resource use and clinical consequences of implementing CCE in routine symptomatic practice. **Conclusions**: DanCap is intended to inform decisions about CCE pathway implementation rather than to establish unbiased whole-cohort test sensitivity or specificity. Trial registration: ClinicalTrials.gov NCT06475560; first submitted 20 June 2024 and first posted 26 June 2024. Recruitment began on 27 November 2024; the registry listed the study as recruiting when last updated on 19 March 2026.

## 1. Introduction

Implementation of national colorectal cancer screening programmes and investigation of gastrointestinal symptoms have increased demand for conventional colonoscopy (CC). CC permits biopsy and treatment but is hospital based, may require sedation or analgesia and is experienced as uncomfortable by some patients. Physical, cultural and practical barriers may also deter attendance [1,2,3,4,5,6,7,8]. CCE does not require endoscope intubation or sedation and, after capsule administration, the patient can leave the endoscopy unit while the examination proceeds. It also requires less procedural equipment than CC. These features make CCE attractive as a first-line triage examination, particularly when the expected prevalence of findings requiring treatment is low; however, any practical advantage must be weighed against downstream CC when CCE is incomplete or identifies a lesion requiring biopsy or treatment.

### Prior Evidence, Knowledge Gap and DanCap Contribution

PillCam COLON 2 (Medtronic, Minneapolis, MN, USA, ) is a second-generation CCE system. Comparative trials and systematic reviews have examined diagnostic performance, bowel preparation and patient acceptability [9,10,11,12], and routine CCE services have been implemented in the United Kingdom [13,14]. A large Danish randomized trial has also evaluated CCE as an alternative within the FIT-positive colorectal cancer screening pathway [15]. Thus, existing evidence has primarily addressed diagnostic performance, preparation quality, acceptability, screening and routine service feasibility.

A persistent implementation limitation is that CCE cannot provide biopsy or treatment, and that incomplete examinations, poor cleansing or clinically important findings require subsequent CC. Reported reinvestigation rates may therefore erode both patient and economic advantages [15,16]. The remaining clinical knowledge gap is not simply whether CCE can detect colorectal lesions, but whether a CCE-first strategy can function as a complete diagnostic pathway for symptomatic referrals without transferring excessive burden and cost to downstream colonoscopy.

DanCap addresses this gap by comparing the complete CCE-first pathway with routine CC in symptomatic patients referred from general practice. Pathway cost is the primary outcome, while lesion detection, examination quality, reinvestigation and patient-reported consequences are evaluated as secondary outcomes. An optional exploratory FIT and microbiome substudy examines whether biomarkers may help predict a clinically actionable CCE result, but these tests do not determine the assigned clinical investigation [17,18,19,20,21]. This pathway-level health services focus distinguishes DanCap from paired diagnostic-accuracy studies. The approved trial protocol (version 2.9, 30 July 2024) predates SPIRIT 2025; the present protocol article is therefore reported with reference to SPIRIT 2025 [22], and the economic reporting framework is informed by CHEERS 2022 [23].

## 2. Objectives

### 2.1. Primary Objective

To compare the costs of the CCE-first diagnostic pathway with those of the routine CC pathway in symptomatic patients referred from general practice.

### 2.2. Secondary Objectives

To compare polyp and colorectal cancer detection rates between the two pathways.

To compare pathway quality measures, including examination completion, bowel cleansing quality, complications and reinvestigation.

To compare patient-reported experience and resource consequences captured by the baseline and 14-day questionnaires.

To explore, within consenting CCE participants, whether FIT and stool microbiome findings predict a clinically actionable CCE result.

To compare the routine clinical CCE reading with research AI output as a quality assurance analysis.

## 3. Materials and Methods

### 3.1. Study Design and Setting

DanCap is a single-centre, two-pathway cluster-allocated crossover trial conducted at the Department of Surgery, Odense University Hospital (OUH), Denmark [24]. General practice clinics are the clusters; individual referred patients are the participants. Clinic-level allocation was selected because the intervention is an entire referral pathway implemented through general practice. Individual randomization would require the same clinic to operate two referral routes concurrently, increasing the risk of contamination, referral errors and selective pathway use. The crossover allows each clinic to contribute participants under both pathways and improves control for stable clinic-level characteristics. Different participants are recruited in the two periods, so there is no participant-level biological carry-over; period, allocation sequence and possible operational learning or carry-over are addressed in the analysis and process evaluation. Participants themselves do not cross over and undergo only the pathway assigned at enrolment. The protocol publicly available with the trial record is version 2.9 dated 30 July 2024.

At study initiation, patients referred from GP clinics with odd provider numbers are assigned to CC and patients from clinics with even provider numbers to CCE. After 200 consecutive CCE participants have been included, the clinic assignments cross to the opposite pathway. Provider numbers pre-exist enrolment and their parity was prespecified as the allocation rule; no trial-generated random sequence is produced, and the sequence can therefore be inferred. Accordingly, the present report uses the term cluster-allocated rather than cluster-randomized. The referral area is Funen, Denmark, with approximately 0.5 million inhabitants. ClinicalTrials.gov retains the labels Randomized and Crossover Assignment; however, assignment is determined by the prespecified GP-provider-number parity rather than by chance, and individual participants receive only one pathway.

### 3.2. Participants

All symptomatic patients aged >18 years who are referred by a GP to OUH for an expedited diagnostic lower-gastrointestinal procedure within 2 weeks because of symptoms compatible with neoplastic disease and who can understand the study information and provide oral and written informed consent are eligible for screening. This is therefore a pragmatic symptomatic diagnostic cohort rather than a population screening cohort. A triaging physician assesses the referral, the project secretary checks the exclusion criteria during contact, and a project physician resolves uncertainties. The current ClinicalTrials.gov record contains two exclusions not listed in the publicly posted version—imaging suggestive of a colorectal tumour and clinical suspicion of microscopic colitis requiring biopsy; Table 1 reflects the current registered eligibility criteria. 

**Table 1:** Exclusion criteria in the current registered DanCap pathway.

No.	Criterion
1	Need for inpatient colonoscopy
2	Previous colonoscopy with poor bowel preparation within the preceding 5 years
3	Inability to provide oral and written informed consent
4	History of gastrointestinal stenosis
5	Previous major gastrointestinal surgery resulting in an internal diversion or a stoma
6	Pacemaker or defibrillator
7	Pregnancy or breastfeeding
8	Known allergy to the bowel preparation regimen
9	Severe kidney disease
10	Known chronic constipation
11	Imaging suggestive of a colorectal tumour
12	Clinical suspicion of microscopic colitis requiring biopsy

### 3.3. Recruitment, Consent and Pathway Assignment

After referral triage, potential participants receive an electronic invitation describing DanCap and an upcoming telephone call. The project secretary provides oral information. Interested patients receive the participant information, baseline questionnaire and consent form by post. They are allowed at least 24 h to consider participation. Written informed consent is obtained before trial-related bowel preparation, FIT sampling or endoscopy. Patients who decline return to the routine CC pathway outside the study.

Consenting patients are assigned to CCE or CC according to their GP clinic provider number parity. Because the procedures are visibly different, participants, treating clinicians and procedure readers are not blinded. Because the parity-based sequence is potentially inferable, referral volumes and enrolled baseline characteristics will be summarized by allocation sequence and period to assess whether material differential referral or participation patterns occurred.

Participants may withdraw consent at any time without affecting their subsequent diagnostic care.

### 3.4. CCE-First Pathway

CCE participants are offered an optional stool sample for fecal hemoglobin concentration and microbiome analysis. Participation in DanCap is not conditional on providing this sample. The sample is collected before bowel preparation and does not influence the clinical pathway. The approved protocol specifies collection of approximately 1 g of stool; fecal hemoglobin is measured first, after which the sample is frozen for analysis of microbial DNA to characterize the microbiome. Human DNA is not examined. The microbiome component is therefore metagenomic and is not limited to genus-level diversity. The posted version 2.9 protocol also specified microbial-DNA analysis of residual colonoscopy tissue from both pathways after routine pathological assessment; this ancillary tissue component is separate from the current registered stool-microbiome outcome and will be reported separately if undertaken.

**Figure 1:**
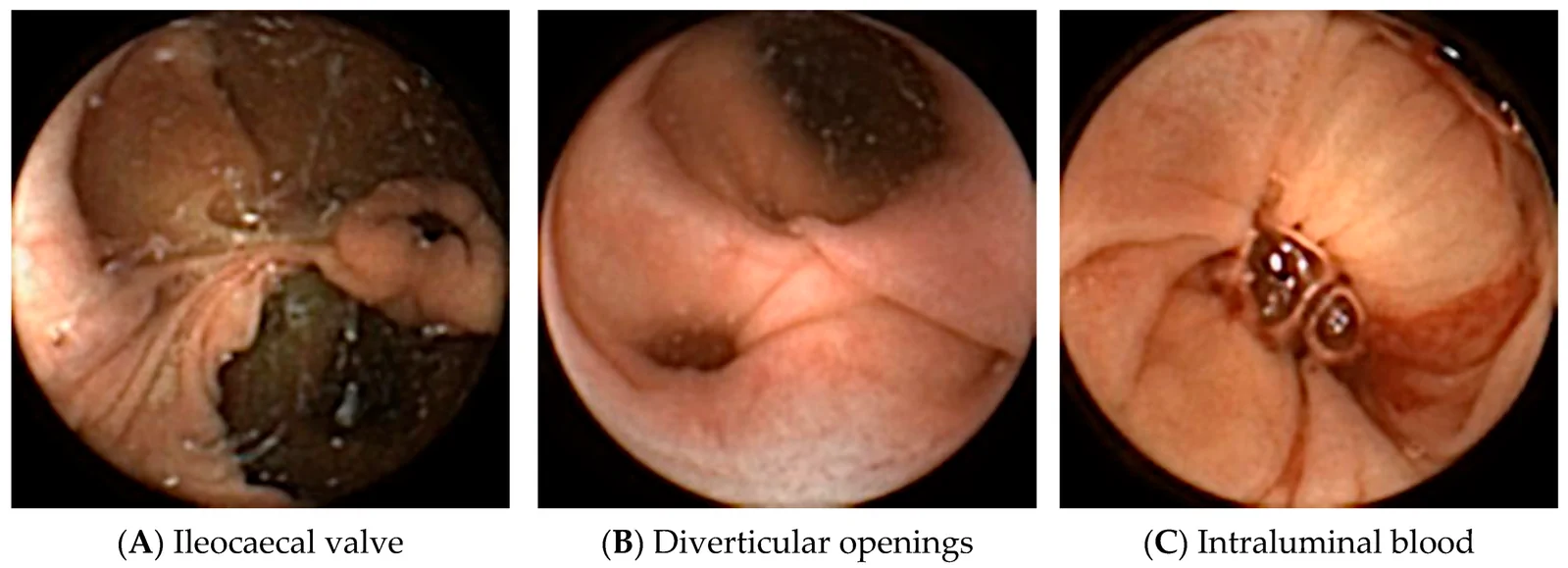

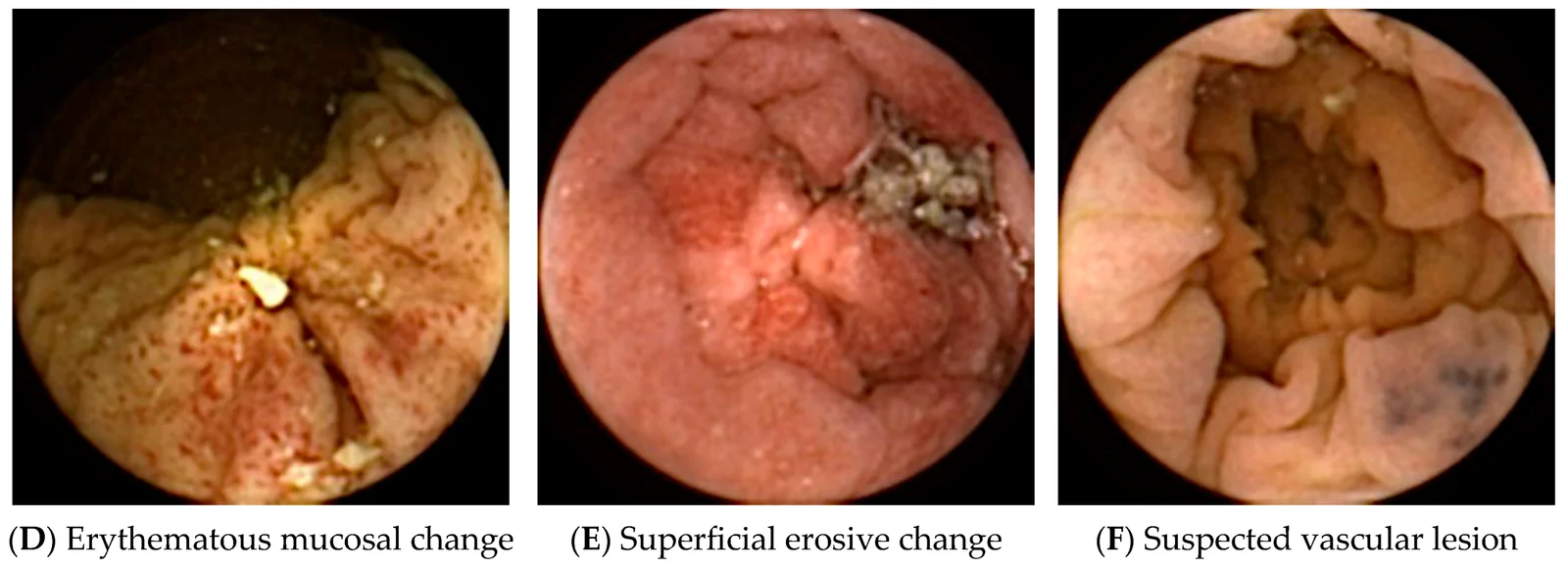
Representative colon capsule endoscopy appearances: (**A**) ileocaecal valve; (**B**) diverticular openings; (**C**) intraluminal blood; (**D**) erythematous mucosal change; (**E**) superficial erosive change; and (**F**) suspected vascular lesion. The frames are included solely to illustrate appearances that may be encountered during clinical capsule reading and do not represent DanCap trial results.

CCE is performed with PillCam COLON 2 (Medtronic, Minneapolis, MN, USA). Participants receive dietary instructions and the preparation/booster regimen in Table 2. Twice weekly, groups of up to five participants visit the hospital clinic. They bring the signed consent form, baseline questionnaire and, when accepted, the stool sample. On the examination day, 2 mg prucalopride is administered before capsule ingestion. Recorder alerts prompt physical activity, chewing gum, two PicoPrep boosters and bisacodyl. Participants return the sensor belt (Medtronic, Minneapolis, MN, USA) and recorder (Medtronic, Minneapolis, MN, USA) when the examination is complete or on the following day, whichever occurs first. Representative CCE appearances that may be encountered during routine clinical reading are shown in Figure 1.

CCE is considered technically complete when the recording has no interruption that prevents evaluation and the ileocaecal valve/caecum is identified, with the capsule excreted within battery life or the anal cushions recorded. Bowel cleansing is graded using the Leighton–Rex scale. An incomplete examination, poor cleansing or a technical failure that prevents satisfactory evaluation leads to CC. For the primary intention-to-treat and cost analyses, these participants remain in the CCE-first pathway; the downstream CC is recorded as reinvestigation and included in CCE pathway resource use and cost.

Suspected cancer or any polyp ≥6 mm leads to diagnostic CC within 2 weeks for biopsy or polypectomy. The ≥6 mm referral threshold is consistent with ESGE/ESGAR guidance [25]. Participants with no neoplastic finding or only polyps <6 mm do not undergo a protocol-mandated early CC. The CCE result is documented in the routine electronic health record and communicated to the participant; thereafter, clinical responsibility returns to routine care, with further investigation when clinically indicated and routine screening when eligible. Any additional lower-gastrointestinal investigation generated by the index diagnostic episode is recorded as pathway reinvestigation; later routine screening is not attributed to the index pathway.

### 3.5. Conventional Colonoscopy Pathway

Participants allocated to CC receive bowel preparation and outpatient colonoscopy according to routine OUH practice and European guidance [26]. The appointment details and instructions are sent electronically, and the preparation kit is sent by post. Findings, interventions, completion, cleansing quality, complications and any subsequent examination are recorded from the routine clinical documentation.

### 3.6. Questionnaires and Follow-Up

Participants in both pathways complete questionnaire Q1 before the procedure and Q2 14 days afterwards. Q1 records baseline information, transport and procedure-related information. Q2 records occupation, recovery and consequences of the procedure. Q2 is distributed through Digital Post. Clinical data and Q1 responses are entered into REDCap; Q2 responses are transferred automatically.

### 3.7. Outcomes

Polyp detection rate (PDR) is a participant-level pathway-yield measure, defined as the proportion of participants with at least one colorectal polyp detected; it is not a per-polyp sensitivity measure. Polyp findings will also be summarized descriptively by the prespecified size categories <6 mm, 6–9 mm and >9 mm. The posted version 2.9 protocol listed NPV, PPV, sensitivity and specificity as a diagnostic-performance outcome, whereas this outcome is not present in the current ClinicalTrials.gov outcome set. The trial does not provide a reference-standard CC for every CCE-negative participant. Consequently, unbiased whole-cohort sensitivity, specificity, PPV and NPV cannot be estimated without verification bias; any diagnostic-performance calculations in verified subsets, if performed, will be exploratory and clearly labelled as such. Pathway-level detection and yield will therefore be reported as the principal comparative clinical measures (Table 3).

**Table 3:** Outcomes and assessment windows.

Class	Outcome	Definition	Time Point
Primary	Pathway cost	Resource use and cost components associated with the index procedure and subsequent investigations generated by that pathway; hospital and patient/societal consequences reported separately	Through completion of the diagnostic pathway
Secondary	Polyp detection rate	Proportion of participants with at least one colorectal polyp detected within the assigned pathway	Diagnostic pathway
Process measure	Colorectal cancer detection	Proportion of participants with colorectal cancer detected within the assigned pathway	Diagnostic pathway
Process measure	CCE completion and cleansing	Completion and Leighton–Rex cleansing assessment as described in Section 3.4	Index CCE
Process measure	CC completion and cleansing	Completion and cleansing quality recorded in the routine colonoscopy report	Index CC
Secondary	Reinvestigation	Additional lower-gastrointestinal examination caused by an incomplete/poor-quality examination or a finding requiring further investigation or treatment	Diagnostic pathway
Process measure	Complications/adverse events	Events documented in routine records and at the 14-day follow-up	Index procedure to Q2
Process measure	Patient-reported consequences	Transport, time and procedural/post-procedural consequences captured by Q1 and Q2	Baseline and day 14
Secondary	FIT and microbiome	Within stool-providing CCE participants, association with a clinically actionable CCE result	Before preparation and diagnostic pathway
Quality assurance	AI quality assurance	Differences between routine clinical CCE reading and AI-assisted review	After routine clinical reading

### 3.8. Participant Timeline

The participants timeline summarized in Table 4 is compliant with the regulations and indications of the danish guidelines regarding diagnostic and treatment of specific gastrointestinal disease. 

**Table 4:** Schedule of enrolment, interventions and assessments.

Activity	Screening	Enrolment	Index/Pathway	Day 14
Referral screening and participant information	X			
Written consent and Q1		X		
Optional stool sample (CCE only)		X		
CCE or CC			X	
Clinical reading and quality outcomes			X	
Additional CC/pathology if indicated			X	X
Q2 and post-procedural consequences				X
Resource-use ascertainment			X	X

### 3.9. Cost–Consequence Analysis

The cost–consequence analysis will present resource use and costs alongside clinical and patient-reported outcomes. Hospital resource inputs include staff time, equipment and materials, CCE reading time, CC procedural staff time, pathology, complications and additional investigations. In the CCE-first arm, any CC generated by an incomplete or poor-quality examination or by an actionable CCE finding is part of the CCE pathway and is included in its resource use and cost. CCE recording duration can be described as a technical process measure where available, but passive capsule transit time is not treated as equivalent to colonoscopy withdrawal or procedure time; the economic analysis focuses on resource-consuming staff and procedural time. Data sources include departmental administrative data, Bookplan timelines, electronic health records, research observations and relevant Danish cost sources, including Behandlingsrådet. Patient transport, time and other consequences recorded in the questionnaires will be reported separately from hospital costs.

Activities introduced only for research—such as recruitment calls, consent administration, research questionnaires, interviews and research meetings—will be excluded from the routine service cost estimate and reported separately when relevant. To improve transferability beyond Denmark, raw resource quantities, such as staff minutes, procedure counts and consumables, will be reported separately from the monetary unit costs applied to those resources. The price year, unit-cost sources and sensitivity analyses will be specified in the final health–economic analysis plan.

### 3.10. Sample Size

The prespecified recruitment target is 800 participants (400 per pathway). Protocol version 2.9 selected this target to support detailed pathway cost estimation across anticipated categories of positive findings and technically unacceptable examinations. The protocol anticipated clinically relevant positive findings in 10–20% and technically unacceptable examinations in 10–15% of participants and sought at least 10 participants in the smallest cost category. It also anticipated that approximately 10% of patients assessed for participation would decline or be ineligible, so approximately 880 records might need to be reviewed to enrol 800 participants.

This feasibility-based target was not derived from a formal comparison of mean pathway cost. No target between-pathway cost effect size or intracluster correlation coefficient (ICC) was assumed, and no design-effect inflation was applied for GP-clinic clustering, unequal cluster sizes, period or crossover. No separate post-enrolment attrition inflation was specified; technically unacceptable or incomplete examinations are pathway outcomes rather than reasons for excluding enrolled participants. The study may therefore have limited statistical power to detect small between-pathway cost differences. Precision for the primary cost difference and secondary detection rate differences will be reported with confidence intervals and interpreted considering the observed cluster-period structure.

### 3.11. Statistical Analysis

A statistical analysis plan will be finalized before database lock. Protocol version 2.9 specified descriptive comparisons, conventional tests for categorical and continuous outcomes, multivariable logistic regression and robust methods to address GP-level clustering. The present SAP framework is a methodological refinement to be finalized before database lock; it does not change the registered primary outcome or the principal registered secondary outcomes. Baseline characteristics and pathway process measures will be summarized descriptively. Primary analyses will follow the intention-to-treat principle according to the clinic-assigned pathway at enrolment, irrespective of downstream investigations or protocol deviations. Thus, for example, an incomplete CCE followed by CC remains analyzed in the CCE-first pathway because that CC is a consequence of the assigned strategy. Estimates will be reported with confidence intervals rather than relying only on *p* values.

Cost will be analyzed with a generalized linear mixed model or other regression model appropriate to the observed cost distribution and link, with pathway, period and allocation sequence as fixed effects and GP clinic as a random intercept. Marginal mean costs and the between-pathway difference will be reported. Alternative distributional assumptions and cluster-level resampling will be considered in sensitivity analyses.

Binary outcomes, including PDR, colorectal cancer detection, completion, adequate cleansing and reinvestigation, will be analyzed with generalized mixed-effects models using pathway, period and allocation sequence as fixed effects and GP clinic as a random intercept. Absolute risk differences and corresponding confidence intervals will be reported. Potential operational carry-over or differential period effects will be examined through the period and sequence terms and, if warranted, in a pathway-by-period sensitivity analysis. Any adjustment for important baseline imbalances will be prespecified in the statistical analysis plan.

Missingness and its reasons will be reported for every outcome. The final SAP will prespecify whether complete-case analysis, multiple imputation or another method is appropriate for each endpoint. FIT and microbiome analyses are restricted to the CCE substudy and are exploratory; high-dimensional metagenomic analyses will require prespecified feature handling and multiplicity control. External clinical risk scores are not part of the prespecified substudy and would only be evaluated in a separately prespecified analysis if the required variables and a clearly defined validated model are available.

### 3.12. Interim Operational Review and Quality Assurance

After 100 CCE participants, the steering group reviews reading quality, completion, adverse events, reinvestigation, complications and clinical workflow. The approved protocol identifies a reinvestigation rate below approximately 25–30% as desirable for patient preference and economy and prespecifies 35% as the upper operational threshold for the interim review. The 35% value is therefore a pragmatic feasibility boundary rather than a statistically derived efficacy stopping rule. If the benchmark is exceeded, the steering group will document the reasons and determine whether workflow modification, protocol amendment, continuation or termination is appropriate.

An expert group performs ongoing quality control of routine CCE readings. AI algorithms developed through the AICE collaboration are applied as an additional quality assurance layer and do not replace the initial clinical reading. AI-only lesions are reviewed and the CCE examination is re-evaluated. When quality control identifies an additional finding, a project physician assesses it, and subsequent management follows the clinical standards specified in the protocol.

### 3.13. Data Management

Clinical and questionnaire data are stored in REDCap hosted by Open Patient data Explorative Network (OPEN). CCE videos and reports are stored in the Gastrointestinal Imaging Database (GAIA), and metagenomic results are held in the approved secure environment. Access is limited to authorized project staff with a research purpose. Data handling follows the EU General Data Protection Regulation and the Danish Data Protection Act.

### 3.14. Patient and Public Involvement

The protocol and participant-facing materials were discussed with the patient representative group associated with the research unit, and corrections and clarifications were made in response to its feedback.

### 3.15. Ethics and Dissemination

The regional ethics committee approved DanCap (reference S-20240043), and written informed consent is obtained before trial procedures. The study is registered at ClinicalTrials.gov (NCT06475560); the study was registered before enrolment, and the registry listed the trial as recruiting when last updated on 19 March 2026. Substantive amendments are handled according to ethics, institutional and registry requirements. Results will be published whether positive, negative or inconclusive.

## 4. Discussion

DanCap evaluates CCE as part of a diagnostic pathway rather than as an isolated test. CCE may avoid some primary colonoscopies, but it cannot provide biopsy or treatment and may generate additional CC after incomplete examinations, poor cleansing or actionable findings. These downstream procedures are deliberately treated as consequences of the CCE-first strategy and are included in the economic and clinical evaluation. This whole-pathway perspective is central to the study’s contribution.

The ≥6 mm threshold for referral to CC is consistent with European CCE management guidance [25]. Participants with smaller polyps do not undergo protocol-mandated early CC, whereas diminutive lesions found during an index CC may be removed during that procedure according to routine practice. This is an intentional difference between the two diagnostic strategies and should be interpreted as a pathway-management and resource consequence rather than as evidence of superior per-polyp detection by either modality. Participants leaving the trial-specific pathway remain under routine clinical responsibility, and further investigation remains available when clinically indicated.

Strengths include implementation within a high-volume clinical service, detailed capture of pathway resource use and patient questionnaires, experienced CCE readers, and the crossover of GP clinics between pathways. Several potential sources of bias nevertheless require consideration. Selection bias is possible because provider number parity is a predictable allocation rule and referring clinicians may infer the current pathway; referral volumes and participant baseline characteristics will therefore be examined by sequence and period. Performance bias cannot be eliminated because CCE and CC are visibly different, but standardized preparation, management thresholds and routine clinical protocols reduce avoidable variation. Detection bias is possible because the modalities and readers differ; ongoing expert quality control and AI-assisted quality assurance provide additional review of CCE readings. Clinic-level allocation reduces contamination from a single practice operating both pathways concurrently. There is no participant-level biological carry-over because different patients are enrolled in each period, although operational learning or workflow carry-over may occur and will be considered through period and sequence effects.

Verification bias is an important limitation. A reference-standard CC is not performed after every negative CCE, so missed lesions cannot be quantified with the certainty of a paired diagnostic accuracy study. This limits estimation of sensitivity and specificity, per-polyp or morphology-specific performance—including performance for laterally spreading lesions—and also constrains inference about clinical safety after a negative CCE. The protocol captures pathway-detected cancer, reinvestigation and short-term adverse events, but it is not designed to estimate post-CCE or interval colorectal cancer over a surveillance horizon comparable with colonoscopy; that question would require longer-term registry-linked follow-up.

Generalisability is also bounded by the pragmatic population and setting. DanCap enrols symptomatic adults referred through a 2-week diagnostic lower-gastrointestinal pathway because of symptoms compatible with neoplastic disease; its findings should not be extrapolated directly to population screening or to patients requiring inpatient or immediately therapeutic colonoscopy. Danish absolute costs may not transfer directly to other healthcare systems. Reporting physical resource quantities separately from Danish unit costs is intended to allow international readers to revalue the pathway using local prices.

## 5. Conclusions

DanCap will compare the costs and clinical consequences of a CCE-first pathway with routine CC in symptomatic adults referred from general practice. Its results will clarify whether reduced use of primary CC is offset by downstream investigation, incomplete examinations or other pathway consequences.

## Figures and Tables

**Table 2:** DanCap CCE bowel preparation and booster regimen.

Day/Stage	Time	Instruction
5 days before	—	Grain-free diet; pause iron tablets
3 and 2 days before	—	Low-fibre diet
Day before	18:00	Plenvu dose 1 in 500 mL water, consumed over approximately 1 h
Day before	19:00	Additional 500 mL water within 30 min; clear liquids thereafter
Examination day	05:00	Plenvu dose 2 in 500 mL water, consumed over approximately 1 h
Examination day	06:00	Additional 500 mL water within 30 min
Examination day	08:00	Prucalopride 2 mg
Examination day	09:00	Capsule ingestion
Recorder alert 0	Device prompted	Light physical activity and chewing gum
Recorder alert 1	Device prompted	PicoPrep 1 sachet in 250 mL water, followed by 2–3 glasses of water
Recorder alert 2	Device prompted	PicoPrep 1 sachet in 250 mL water, followed by 2–3 glasses of water
Recorder alert 3	Device prompted	Bisacodyl 10 mg

## Data Availability

No trial outcome data are presented in this protocol article. Availability of deidentified trial data after study completion will be governed by participant consent, Danish data protection law, ethics approval and institutional agreements.

## Abbreviations

The following abbreviations are used in this manuscript:

AI	Artificial Intelligence
AICE	Artificial Intelligence Capsule Endoscopy
CC	Conventional Colonoscopy
CCA	Cost–Consequence Analysis
CCE	Colon Capsule Endoscopy
CRC	Colorectal Cancer
FIT	Fecal Immunochemical Test
GAIA	Gastrointestinal Imaging Database
GDPR	General Data Protection Regulation
GP	General Practitioner
OPEN	Open Patient data Explorative Network
OUH	Odense University Hospital
PDR	Polyp Detection Rate
SAP	Statistical Analysis Plan
SPIRIT	Standard Protocol Items: Recommendations for Interventional Trials
